# Establishing an optimal diagnostic criterion for respiratory sarcopenia using peak expiratory flow rate

**DOI:** 10.1007/s40520-024-02765-z

**Published:** 2024-05-23

**Authors:** Yerim Do, Youngeun Lim, Jiyoun Kim, Haneul Lee

**Affiliations:** 1 Graduate School, Department of Physical Therapy, College of Health Science, Gachon University, Incheon, 21936 Korea; 2 Department of Exercise Rehabilitation, Gachon University, Incheon, 21936 Korea; 3 Department of Physical Therapy, Gachon University, Incheon, 21936 Korea; 4 Department of Physical Therapy, College of Health Science, Gachon University, 191 Hambakmae-ro, Yeonsu-gu, Incheon, 21936 Korea

**Keywords:** Respiratory sarcopenia, Peak expiratory flow rate, Cut-off point, Sensitivity, Specificity

## Abstract

**Background:**

The skeletal muscle changes as aging progresses, causing sarcopenia in the older adult population, which affects the respiratory muscles’ mass, strength, and function. The optimal cut-off point of peak expiratory flow rate (PEFR) for respiratory sarcopenia (RS) diagnosis in accordance with sarcopenia identification is needed.

**Aim:**

To establish an optimal cut-off point of PEFR for RS diagnosis in community-dwelling Asian older women.

**Methods:**

Sarcopenia diagnostic indicators were evaluated according to the Asian Working Group for Sarcopenia 2019 (AWGS) criteria. The respiratory parameters composed of respiratory muscle strength and respiratory function were evaluated by assessing maximal inspiratory pressure (MIP), percent predicted forced vital capacity (Pred FVC), and PEFR.

**Results:**

A total of 325 community-dwelling older women were included in this study. PEFR was negatively associated with RS (OR: 0.440; 95% CI: 0.344–0.564). The area under the curve (AUC) of PEFR was 0.772 (*p* < 0.001). The optimal cut-off point of PEFR for RS diagnosis was 3.4 l/s (sensitivity, 63.8%; specificity, 77.3%). Significant differences were found between the robust, possible sarcopenia, sarcopenia, and RS groups in terms of both sarcopenia diagnostic indicators and respiratory parameters (*p* < 0.05).

**Conclusions:**

The cut-off point of PEFR can be used as a reasonable standard for RS diagnosis. This study finding can serve as a cornerstone for developing concrete criteria of RS in older women, supporting clinical judgment, which is crucial for providing appropriate treatment through accurate diagnosis.

## Introduction

Sarcopenia, which is a progressive syndrome defined as the age-related loss of skeletal muscle mass with loss of muscle strength and/or physical performance is often experienced by the older adult population [1–4]. As sarcopenia is associated with physical inactivity, decreased activities of daily living, falls, long-term hospitalization, economic burden, and mortality, it is believed to have a profound effect on the health of the older adult population [3, 5–7]. Respiratory muscles such as the diaphragm are known to be affected by sarcopenia as other muscles of the upper and lower extremities [8]. In addition, the indicators used for evaluating the respiratory system such as diaphragmatic mobility, maximum respiratory pressures, and peak expiratory flow rate (PEFR) were shown to be affected [4, 8, 9].

Dysfunctions of the respiratory system can cause serious health-related problems including inadequate ventilation followed by inappropriate removal of airway secretions, increased incidence of comorbidities, and poor cognitive performance [10, 11]. Specifically, the decreased muscle mass and strength of the respiratory muscles reduce the ability to carry oxygen, causing skeletal muscle fatigue easily with reduced exercise tolerance [12, 13]. This can adversely affect gait, activities of daily living, and quality of life leading the older adult population to be more vulnerable to disease [12–14]. Considering the possible impacts of muscle weakness and functional degradation related to the respiratory system in the older adult population, the need to develop an accurate definition and concrete criteria for this state has been suggested [12, 15]. In this regard, the definition of respiratory sarcopenia (RS) was recently established as the concurrent occurrence of declines in respiratory indicators and sarcopenia by several studies, but there is a lack of consensus regarding the definition [12, 15].

The criteria for defining loss of respiratory muscle strength and function are presented through maximal inspiratory pressure (MIP) and percent predicted forced vital capacity (Pred FVC) [13, 16–19]. Although Pred FVC has been suggested as an indicator of respiratory function, PEFR should be considered as an alternative indicator. The changes in mechanical properties of the chest wall, respiratory muscles, and joints related to the respiratory system during aging can cause a decrease in FVC even though respiratory muscle strength is maintained [19]. In contrast, PEFR depends on an expiratory effort and respiratory muscle strength and is minimally affected by the compliance of the respiratory system when observed at initial exhalation [19, 20]. Additionally, PEFR provides safe measurements without increasing blood pressure and has been reported to be associated with skeletal muscle mass, sarcopenia, and respiratory muscle strength [15, 21]. Hence, this study aimed to establish the cut-off point of PEFR for RS diagnosis as a criterion of respiratory function in consideration of the presence of sarcopenia.

## Materials and methods

### Study design

This study was an observational cross-sectional, double-blinded design. This manuscript follows the Standards for Reporting of Diagnostic Accuracy Studies (STARD) guidelines. All procedures in this study followed the guidelines of the Declaration of Helsinki and were approved by the Institutional Review Board of Gachon University (1044396-202204-HR-082-02). Prospective recruitment and evaluation were conducted from July 2022 to May 2023. The participants were fully informed about the purpose and procedure of this study before starting the measurements and signed a written informed consent form.

### Participants

A total of 390 community-dwelling Asian women over 65 years participated in this study. The participants were recruited through senior citizen’s centers and welfare centers in two metropolitans. They were enrolled using a non-probability consecutive sampling method. The exclusion criteria were a history of pulmonary diseases, neuromuscular diseases, thoracic surgery, heavy smokers (more than 20 packs per year), inappropriate bioimpedance analysis testing due to the presence of pacemakers, or surgeries involving the insertion of metallic substances, and difficulties in following measurement instructions [8, 22].

The sample size was calculated by using Med Calculator software 12.3.0 (MedCalc Software Babv, Belgium). The alpha error probability was set at 0.05 with a power of 0.80. No previous study has provided the area under the curve (AUC) of diagnostic variables for RS prediction, but a strong effect size of PEFR (r-square = 0.797) for RS prediction has been reported [15]. Hence, a poor diagnostic ability (AUC = 0.690) was considered for sample size estimation. The ratio of sample sizes in the negative and positive groups was set at 547 and 134, respectively according to a previous study that reported the predictive accuracy of PEFR [15]. As a result, the minimum number of participants required to establish a cut-off point for RS diagnosis was 117. However, to achieve a more accurate diagnosis, as many subjects as possible were recruited.

### Test methods

A positive RS result was defined as sarcopenia accompanied by a loss of respiratory muscle strength and/or a decline in respiratory function. Despite various definitions of RS have been proposed in previous studies, a definition considering the presence of sarcopenia rather than one disregarding it was selected to compensate for the absence of low respiratory muscle mass criteria. The PFER value from the spirometer was used as the index test.

The reference standard representing the final diagnosis of RS complies with existing criteria. The presence of sarcopenia was defined according to the Asian Working Group for Sarcopenia 2019 (AWGS) criteria [5]. The predicted value of MIP adjusted by age and sex was calculated using the equation presented by Nishimura et al. (131 − 0.76 × age for men and 102 − 0.69 × age for women, respectively), and was used as a criterion for judging respiratory muscle weakness [13]. Participants were defined as having loss of respiratory muscle strength when their MIP was lower than the predicted MIP [13]. Pred FVC of less than 80 was considered a loss of respiratory function [17]. The researchers and participants were blinded to the results of the index test and reference standard test.

### Study procedure

After arriving at the laboratory, age, sex, smoking status, exercise habits, and medical history were collected using a self-administered form, and instructions for testing were provided prior to the beginning of the measurements. Height, weight, skeletal muscle mass, handgrip strength, physical performance, respiratory muscle strength, and respiratory function were measured. Confirmation of participant eligibility in relation to the presence of disease according to the exclusion criteria or the state of smoking was identified prior to the day of participation.

After participants performed all measurements, they were divided into four groups as follows: (1) robust (no sarcopenia and no loss of respiratory muscle strength and function); (2) possible RS (only respiratory strength and/or function loss without sarcopenia); (3) sarcopenia (sarcopenia only appeared with no decrease in respiratory indicators); (4) RS (both sarcopenia and loss of respiratory indicators were observed).

## Measurements

### The presence of sarcopenia through the AWGS

#### Muscle mass

Bioelectrical impedance was measured using an Inbody 120 (Inbody Corp., Seoul, Korea). Participants were instructed to wear as light clothing as possible and to be a barefoot, and a tapeline was used to measure their height. Appendicular skeletal muscle mass (ASM) was calculated using the following equation: ASM (kg) = 0.244 × body weight (kg) + 7.8 × height (m) + 6.6 × gender (1 for men and 0 for women) − 0.098 × age (years) + race (0 for whites, 1.4 for blacks and − 1.2 for Asians) − 3.3 [9, 23–25]. The muscle mass index was estimated by dividing the ASM value by height squared [25].

#### Muscle strength

A Smedley-type handheld dynamometer (Fabrication Enterprises Inc., Elmsford, NY, USA) was used to measure hand grip strength [26]. Participants were encouraged to provide their maximal effort for 5 s with both dominant and non-dominant hands twice for each alternately while maintaining a standing posture with fully extended elbows. The best performance was used for analysis. Thirty seconds of rest period was given between each measurement to prevent muscle fatigue [26, 27].

#### Physical performance

The short physical performance battery (SPPB) protocol, with a total score ranging from 0 to 12, was used as a useful tool for measuring functional state and physical performance. For the balance test, participants were evaluated based on their ability to maintain the side-by-side stand, semi-tandem stand, and tandem stand postures for a certain duration. Gait speed was assessed by measuring the time taken to walk 4 m at their usual walking speed, while lower limb strength was evaluated based on the time they needed to complete the 5-times chair stand tests [28, 29].

### The respiratory muscle strength

MIP was measured as the strength of the respiratory muscles using POWERbreathe K5 (Power Breathe International, Warwickshire, United Kingdom). Participants were asked to slowly exhale and inhale with their maximal effort three times in the sitting position following the guidelines published by the American Thoracic Society (ATS) after observing the researcher’s demonstration [30, 31]. The highest values were recorded for the analysis. To ensure reproducibility, the variation between the two highest values should be less than 10%. A maximum of 5 trials could be performed if the results were not acceptable [9, 31].

### The respiratory function

Pony FX (COSMED Inc., Rome, Italy) was used to assess the FVC, forced expiratory volume in one second (FEV_1_), and PEFR according to ATS instructions [18]. Following the researcher’s demonstration, participants were informed to perform the test with their maximal efforts in a sitting position, and the highest value among the three trials was recorded. Up to five additional trials were performed if the results were unacceptable [18].

### Statistical analysis

Statistical analyses were performed using Statistical Product and Service Solutions (IBM Corp, Armonk, NY, USA) version 23.0 for Windows and Med Calculator software. Categorical data are presented as numbers and percentages whereas continuous data are presented as mean ± standard deviation (SD). The Kolmogorov-Smirnov test was used for the assumption of normality of the data, and all data met the assumption. Pearson’s correlation was used to verify the relationship between the RS diagnostic variable and sarcopenia diagnostic indicators and respiratory parameters. The correlation coefficients, denoted as r values, are considered to indicate small, medium, and large effects at 0.10, 0.30, and 0.50, respectively [32]. Binary logistic regression was used to evaluate the predictive probability of PEFR for the RS diagnosis. Pearson’s chi-squared test for the cross-tabulation was used to assess sensitivity and specificity of diagnostic criteria for RS, involving a comparison of observed versus expected frequencies in a cross-tabulation. This determined the adequacy of the diagnostic criterion in differentiating between RS-positive and RS-negative cases based on PEFR. The AUC representing the diagnostic accuracy was calculated using receiver operating characteristic (ROC) curve analysis, and the DeLong method was used to compare the diagnostic abilities of the respiratory parameters. The Kruskal-Wallis rank test with Bonferroni-corrected post-hoc tests was conducted for the comparison of sarcopenia diagnostic indicators and respiratory parameters among the robust, possible sarcopenia, sarcopenia, and RS groups. Statistical significance was set at 0.05.

## Results

**Fig. 1:**
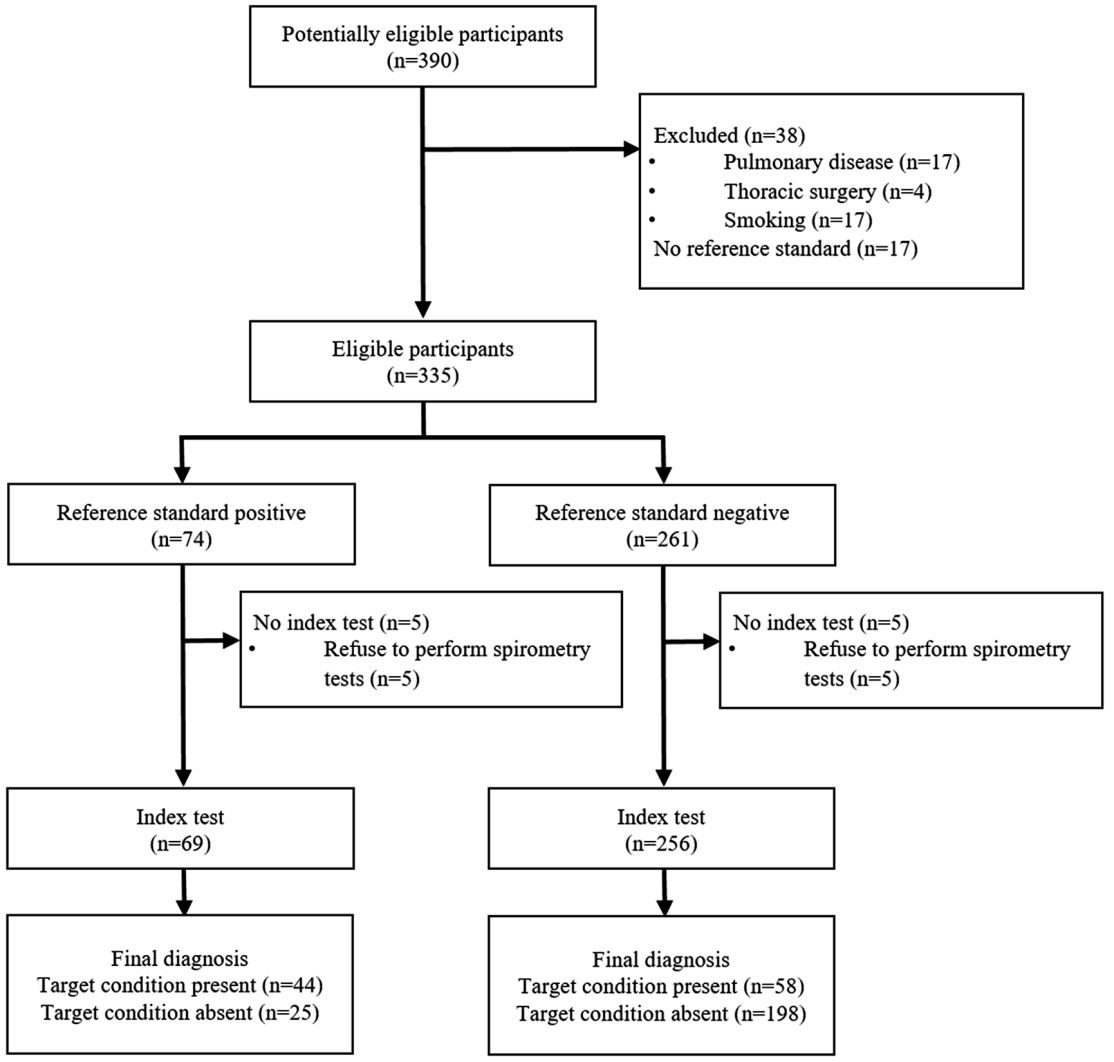
STARD diagram of the participants

A total of 325 women who performed all measurements were included in the analysis (Fig. 1). There were no adverse events that occurred while performing the index test or the reference standard test. However, about 25 individuals had difficulty with the spirometry test, especially with maintaining exhalation for more than 6 s. Each participant was allowed a maximum of five attempts to ensure accurate measurements of respiratory parameters, facilitating them in maintaining an adequate duration of exhalation to complete the test. The general characteristics of the participants are described in Table 1. The mean age of participants was 76.17 ± 7.12, and the mean PEFR was 4.18 ± 1.47.

**Table 1:** General characteristics of the participants

	Older Women (*n* = 325)
Age (years)	76.17 ± 7.12
Height (cm)	151.00 ± 6.24
Weight (kg)	56.36 ± 8.16
BMI (kg/m^2^)	24.75 ± 3.55
ASM (kg/m^2^)	5.93 ± 0.90
Grip strength (kg)	19.75 ± 5.14
SPPB (score)	9.64 ± 2.56
MIP (cmH_2_O)	40.61 ± 16.40
FVC (l)	2.12 ± 0.51
FEV_1_ (l)	1.68 ± 0.42
PEFR (l/s)	4.18 ± 1.47

Abbreviation: BMI, body mass index; ASM, appendicular skeletal muscle mass; SPPB, short physical performance battery; MIP, maximal inspiratory pressure; FVC, forced vital capacity; FEV_1_, forced expiratory volume in one second; PEFR, peak expiratory flow rate

There were significant positive correlations between PEFR and sarcopenia diagnostic indicators including ASM (*r* = 0.238, *p* < 0.001), grip strength (*r* = 0.465, *p* < 0.001), and SPPB (*r* = 0.496, *p* < 0.001). In addition, strong correlation coefficients were found between PEFR and respiratory parameters, including MIP (*r* = 0.665, *p* < 0.001), FVC (*r* = 0.654, *p* < 0.001), and FEV_1_ (*r* = 0.745, *p* < 0.001). Binary logistic regression showed that PEFR was significantly associated with RS (OR: 0.440; 95% CI: 0.344–0.564), indicating that an increase by one unit of PEFR decreased the probability of RS by 56%. The cut-off point of the PEFR as a criterion for predicting RS had significant results (*p* < 0.001). The optimal cut-off point of PEFR was 3.4 l/s with an AUC of 0.772 (95% CI: 0.713–0.831), indicating moderate accuracy. Table 2 shows the index test results over the reference standards for RS diagnosis. A significant relationship was observed between the index test and the reference standard test (*p* < 0.001). Sensitivity and specificity were 0.638 and 0.773, respectively. The Youden index was 0.411.

**Table 2:** Cross-tabulation of the index test with the reference standards

		Index test		
		RS Negative	RS Positive	Total
Reference standards	RS Negative	198 (77.3%)	58 (22.7%)	256 (100%)
	RS Positive	25 (36.2%)	44 (63.8%)	69 (100%)
	Total	223 (68.6%)	102 (31.4%)	325 (100%)
χ^2^ (*p*)	42.658 (< 0.001)			

Abbreviation: RS, respiratory sarcopenia

**Fig. 2:**
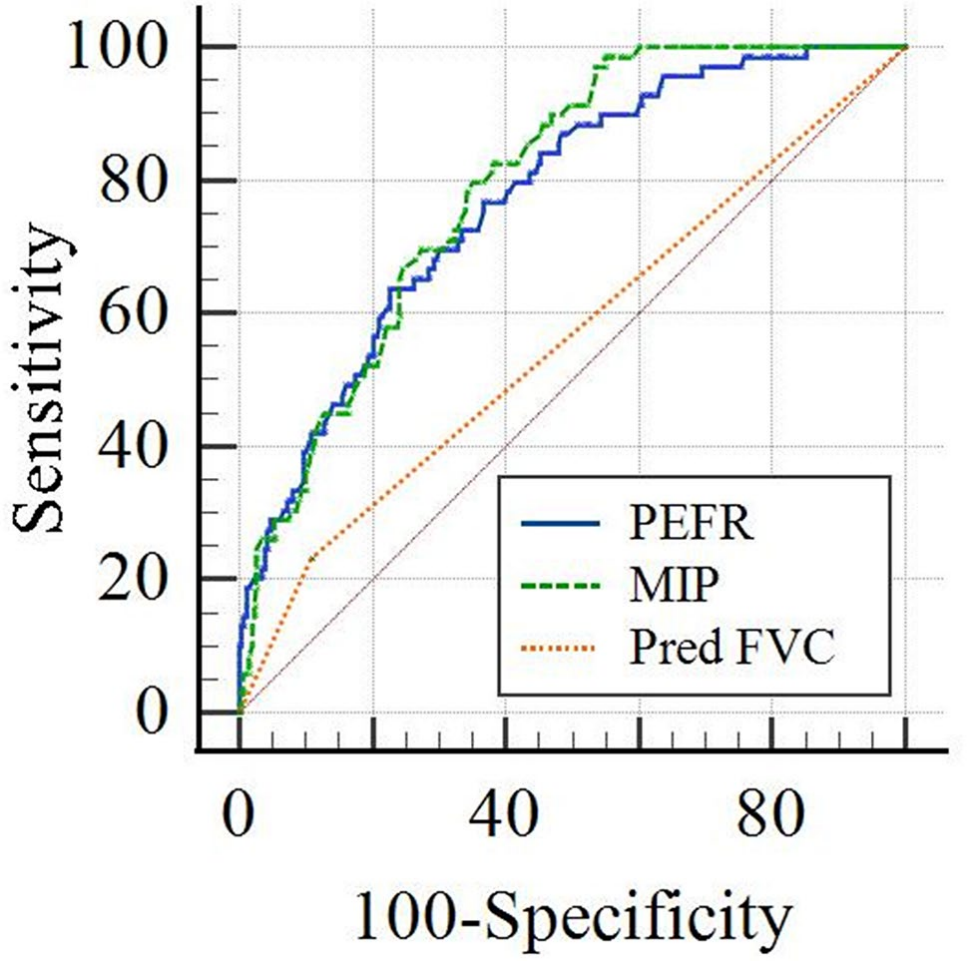
Pairwise comparison of ROC curves of PEFR with MIP, and Pred FVC. Abbreviations: PEFR, peak expiratory flow rate; MIP, maximal inspiratory pressure; Pred FVC, percent predicted forced vital capacity

Table 3 describes the difference between the diagnostic ability of PEFR (AUC = 0.772) and MIP (AUC = 0.792) and Pred FVC (AUC = 0.561). There was no significant difference in AUC values between PEFR and that of MIP (*p* > 0.05) while there was a significant difference with that of Pred FVC (*p* < 0.001) (Fig. 2).

**Table 3:** Comparison of AUC between the ROC curve of PEFR and that of MIP and Pred FVC

	AUC	95% CI	*p*
PEFR (l/s)	0.772	0.723–0.817	
MIP (cmH_2_O)	0.792	0.743–0.835	0.464^a^
Pred FVC (%)	0.561	0.505–0.616	< 0.001^b^

Abbreviations: AUC, area under the curve; CI, confidence interval; PEFR, peak expiratory flow rate; MIP, maximal inspiratory pressure; Pred FVC, percent predicted forced vital capacity a: Comparison of AUC between the ROC curve of PEFR and MIP b: Comparison of AUC between the ROC curve of PEFR and Pred FVC

According to the reference standards, participants were divided into four groups. Differences in sarcopenia diagnostic indicators and respiratory parameters between the four groups were described in Table 4.

**Table 4:** Measurement values of the participants compared by groups

Variables	Robust (*n* = 68)	Possible RS (*n* = 181)	Sarcopenia (*n* = 7)	RS (*n* = 69)	*P* (Bonferroni)
ASM (kg/m^2^)	6.10 ± 0.11	6.24 ± 0.06	5.26 ± 0.14	5.01 ± 0.06	< 0.001 / 0.024 / 0.004 (a, b > d) / (a > c) / (b > c)
Grip strength (kg)	22.41 ± 0.50	20.55 ± 0.36	19.51 ± 1.18	14.99 ± 0.48	< 0.001 (a, b > d)
SPPB (score)	10.90 ± 0.23	10.02 ± 0.17	9.86 ± 0.55	7.38 ± 0.31	0.032 / < 0.001 (a > b) / (a, b > d)
MIP (cmH_2_O)	63.75 ± 1.34	36.01 ± 0.77	57.71 ± 2.23	28.14 ± 1.18	< 0.001 / 0.002 (a, b,c > d, a > b) / (b > c)
FVC (l)	2.52 ± 0.05	2.07 ± 0.03	2.42 ± 0.17	1.81 ± 0.06	< 0.001 / 0.003 / 0.024 (a > b,d) / (b > d) / (c > d)
FEV_1_ (l)	2.00 ± 0.04	1.66 ± 0.03	1.87 ± 0.16	1.38 ± 0.05	< 0.001 / 0.041 (a > b > d) / (c > d)
PEFR (l/s)	5.35 ± 0.16	4.13 ± 0.09	5.19 ± 0.46	3.07 ± 0.15	< 0.001 / 0.001 (a > b > d) / (c > d)

Abbreviations: RS, respiratory sarcopenia; ASM, appendicular skeletal muscle mass; SPPB, short physical performance battery; MIP, maximal inspiratory pressure; FVC, forced vital capacity; FEV_1_, forced expiratory volume in one second; PEFR, peak expiratory flow rate

## Discussion

As the aging population increases, various studies have been conducted on sarcopenia. However, studies on RS, which is a more specific concept of sarcopenia recently introduced, are insufficient. Similar to the criteria outlined for diagnosing sarcopenia were based on muscle mass, strength, and function, specific criteria for each aspect of respiratory muscles need to be established for an accurate diagnosis of RS as well. In previous studies, the RS diagnostic criteria were presented through MIP representing respiratory muscle strength and Pred FVC for respiratory function [12]. Although PEFR was also used to define RS in a previous study, the presence of sarcopenia was not addressed as they only used PEFR for RS determination. As a result, 16.82% of non-RS people were found to have sarcopenia in their study [15]. Thus, establishing a cut-off point for RS diagnosis using PEFR with the process of identifying sarcopenia is required. This study aimed to establish an optimal PEFR cut-off point for RS diagnosis in older women and we hypothesized that the PEFR could be utilized as indicators of RS. Our results showed a significant cut-off point of PEFR for the identification of RS in older women, with PEFR significantly correlated with both sarcopenia diagnostic indicators and respiratory parameters. Furthermore, higher PEFR is shown to be associated with a decrease in RS incidence, indicating the capabilities of PEFR for respiratory function assessment in RS diagnosis. The reported cut-off point of PEFR for RS diagnosis in the present study was 3.4 l/s with moderate accuracy in older women. The cut-off point of PEFR in our study was lower than that of a previous study developed to diagnose sarcopenia. This is presumably because RS in this study refers to a condition in which sarcopenia is accompanied by decreased respiratory muscle strength and/or respiratory function [21]. To estimate diagnostic ability, the AUC of PEFR (AUC = 0.772) was compared with that of MIP (AUC = 0.792) [13], and Pred FVC (AUC = 0.561) [17]. The predictive performance of PEFR was numerically lower than that of MIP, but no significant differences were found while it was significantly higher than Pred FVC.

No previous studies have compared the diagnostic ability of respiratory parameters for RS diagnosis. However, regarding the ability to diagnose sarcopenia, Kera’s model, which used only PEFR as a criterion, was compared with the Japanese Association of Rehabilitation Nutrition (JARN) model using FVC and sarcopenia diagnostic indicators [12, 19]. Although indicators related to sarcopenia were not included in Kera’s model, a higher sensitivity was observed than in the JARN model, indicating the superiority of the PEFR-based model in diagnosing sarcopenia [19]. The aging-related changes in mechanical properties of the chest wall, respiratory muscles, and joints can reduce FVC despite preserved respiratory muscle strength. This implies the potential impact of the association between FVC and compliance of the respiratory system when used as a diagnostic indicator, thereby supporting PEFR as a more reasonable indicator of respiratory function [19]. Unlike PEFR, FVC is influenced by the mechanical properties of the chest wall, respiratory muscles, and joints that changes due to aging. This indicates that FVC may decrease even if respiratory muscle strength is maintained, which may have affected the sensitivity of the test [19]. The failure of participants to perform 6 s of exhalation completely during the spirometry test within a maximum of 5 trials is an additional aspect to consider because it may cause an underestimated FVC, leading to an overestimation of the FEV_1_/FVC ratio [33].

In this study, we observed significant differences in sarcopenia diagnostic indicators and respiratory parameters in a comparison between groups, supporting the validity of PEFR in determining the RS. A decrease in respiratory muscle strength and/or function was observed in 55.7% of the participants, while sarcopenia was observed in 2.2%. In addition, the loss of respiratory parameters was determined in 90.8% of the participants with sarcopenia. Similarly, a previous study by Morisawa et al., also showed that almost all participants with sarcopenia had weakness in respiratory muscles. Therefore, it is necessary to consider the possibility that sarcopenia may be preceded by decreased respiratory parameters [13]. This can be explained by the respiratory metabolic reflex mechanism, indicating that the decrease in respiratory muscle mass and strength degrades the oxygen-carrying capacity. This leads to decreased exercise tolerance, which consequently causes the loss of skeletal muscle mass and strength [12, 13, 34]. Several studies have verified that impairments in respiratory parameters are associated with the development of sarcopenia, but studies to identify whether decreased respiratory function contributes to RS development are lacking. Thus, longitudinal studies are required [13, 34, 35].

This was the first study to establish the cut-off point and diagnostic indices including sensitivity and specificity of PEFR in community-dwelling older women. However, some limitations need to be addressed. The primary limitation is the study’s focus on older women, which may not generalize to older men due to gender-specific differences in sarcopenia and the respiratory system evaluations. Moreover, the cross-sectional design limits the ability to infer casual relationships between respiratory function influenced in causing RS development. While the present study offers valuable insights, its generalizability is restricted, particularly concerning individuals with chronic conditions which could influence respiratory function. Future longitudinal studies, incorporating both genders and considering chronic conditions like cardiovascular disease, are warranted to address the issue of generalizability. Additionally, incorporating other measures of physical function, such as the six-minute walk test, could provide a more comprehensive understanding of the relationship between physical function and RS, further enriching the diagnostic criteria for RS.

The findings of our study demonstrate the importance of clinical implications as providing a standard for RS diagnosis using PEFR in older women. It is an accessible method that enables the development of an appropriate treatment strategy through accurate diagnosis. The adverse events such as hypoxic stress, cachexia, decreased activity, and lower quality of life that can occur due to undiagnosed and untreated RS can be prevented [12].

## Conclusions

The PEFR of 3.4 l/s can serve as a significant predictor for RS. Associations of PEFR with both sarcopenia diagnostic indicators and respiratory parameters were observed in this study. Furthermore, differences in the evaluation indices between the robust, possible sarcopenia, sarcopenia, and RS groups were also determined, supporting the validity of PEFR in diagnosing RS. Overall, the results of this study may contribute to the development of concrete criteria for RS in older women, which is crucial for its accurate diagnosis and providing appropriate treatment. The criterion suggested by this study can be used as a reasonable standard for supporting clinical judgment.

## Data Availability

Data are available upon reasonable request.
